# Association between Neutrophilic Granulocyte Percentage and Diabetes Mellitus in Cushing's Syndrome Patients: A Cross-Sectional Study

**DOI:** 10.1155/2021/9536730

**Published:** 2021-11-29

**Authors:** Baomin Wang, Yumei Yang, Haoyue Yuan, Xiaomu Li

**Affiliations:** Department of Endocrinology and Metabolism, Zhongshan Hospital, Fudan University, Shanghai 200032, China

## Abstract

**Background:**

Glucose metabolism is frequently impaired in patients with Cushing's syndrome (CS) due to chronic exposure to excess glucocorticoids. Inflammation plays an essential role in the pathophysiology of diabetes mellitus (DM). The present study aimed to investigate the potential associations of inflammatory blood cell parameters, including white blood cell (WBC) count, neutrophil count, neutrophilic granulocyte percentage (NEUT%), lymphocyte count (LYM), and lymphocyte proportion (LYM%), with diabetes mellitus in Cushing's syndrome patients.

**Materials and Methods:**

The cross-sectional study was conducted in Zhongshan Hospital of Fudan University, China. A total of 150 patients with Cushing's syndrome were retrospectively screened from 2017 to 2019. The demographic data, clinical data, and blood samples (lipids, adrenal, glucose, and inflammatory blood cell parameters) were recorded. Statistical analyses were carried out by using the SPSS software package, version 13.0.

**Results:**

In this study, the prevalence of diabetes mellitus was 38.7% in patients with Cushing's syndrome. Patients with DM had higher WBC, neutrophil, NEUT% levels than patients without DM (*p* < 0.05). As the NEUT% increased, a stepwise increase in glucose and glycated hemoglobin (HbA1c) level was observed. In addition, in the multivariate logistic regression, NEUT% was a significant independent risk factor for DM, regardless of gender, age, body mass index (BMI), and triglyceride and 12 midnight cortisol (12 MN cortisol) level (OR = 2.542, 95% CI 1.337–4.835, *p* < 0.001).

**Conclusions:**

In conclusion, elevated NEUT% level was linked to diabetes in patients with Cushing's syndrome. The neutrophilic granulocyte percentage may be referred to as a new predictor for diabetes in Cushing's syndrome patients.

## 1. Introduction

Cushing's syndrome (CS) is a set of clinical symptoms caused by long-term exposure to excessive glucocorticoids (GCs) [1]. It was first described in 1912, with diverse clinical features and laboratory examination findings [2], such as obesity, hypertension, diabetes, hyperlipidemia, hirsutism, osteoporosis, and hypokalemia [3, 4]. Due to the insidious features, most patients experience incorrect diagnosis and delayed initiation of treatment, which lowers the disease detection rate. Accurate diagnosis and timely treatment can greatly reduce the disability and mortality rates of patients suffering from Cushing's disease (CD) [5].

Impaired glucose metabolism is frequently encountered in CS [6]. Diabetes mellitus is a joint complication of chronic exposure to glucocorticoids, and it can contribute to increased morbidity and mortality in CS patients [7]. Identifying and correcting conventional risk factors can prevent and delay type 2 diabetes (T2DM) during the early stages of this condition. However, the exact pathophysiology of DM in CS patients remains elusive. Whether there are interconnections between the two and what type of relationship exists are still worth exploring. Therefore, it is still essential to identify other common risk factors for DM to prevent diabetes in the population with Cushing's syndrome.

Notably, leukocytosis is often referred to as a laboratory finding in CS patients. Molecular studies have established that glucocorticoid receptors are expressed in the white blood cell (WBC), while undue secretion of glucocorticoids in CS patients led to activation and increase of leukocytes. As first set out in the early 1940s, elevated WBC counts were positively correlated with Cushing's syndrome. The more severe the symptoms of Cushing's disease, the higher the WBC count. De La Blaze et al. [8] found that the white blood cell count of 10 patients with Cushing's disease was greater than normal subjects, with a higher number of polymorphonuclear (PMN) cells and a lower number of lymphocytes. In addition, previous studies have shown that chronic inflammation plays a crucial role in the pathophysiology of T2DM [9–11]. Many hemogram parameters can interact with the pathological process of diseases characterized by significant or mild inflammation. Several hematological markers have already been reported to be associated with diabetes mellitus. These included red blood cell distribution width (RDW) [12], mean platelet volume (MPV) [13], neutrophil count to lymphocyte count ratio (NLR) [14], platelet count to lymphocyte count ratio (PLR) [15], and MPV to lymphocyte count ratio (MLR) [16]. The association between these indicators and diabetes has been well established. The percentage of neutrophilic granulocyte could also be one of these hematological indices. However, to our knowledge, the association between NEUT% and T2DM with Cushing's syndrome has not been studied. The white blood cell status in Cushing's diabetic patients is not clearly understood. There are also no relevant and accurate data reports on the prevalence of this finding in patients.

The present study explores the potential association between inflammatory blood cell parameters, especially the NEUT%, with type 2 diabetes in 150 Cushing's disease patients.

## 2. Research Design and Methods

### 2.1. Ethics, Consent, and Permission

All procedures were performed in compliance with the Helsinki Declaration. The present study was approved by the Zhongshan Hospital Ethics Committee, Fudan University, China. Written informed consent was collected from each participant.

### 2.2. Study Population

A retrospective study of Cushing's syndrome patients from the Department of Endocrinology, Zhongshan Hospital, from 2017 to 2019 was conducted. All the participants complied with the diagnostic criteria of CS based on the Endocrine Society guidelines [17]. Exclusion criteria included subclinical CS (e.g., serum cortisol levels of ≥1.8 *μ*g/dL after an overnight 1 mg DST without symptoms of CS) [18], renal insufficiency with an estimated glomerular filtration rate (eGFR) of <30 mL/min/1.73 m^2^, Cushing's disease, adrenal carcinoma, and bilateral adenoma. In addition, patients also were excluded from the present study for any of the following reasons: (1) acute infectious disease with the blood cell test two weeks ago; (2) history of parasite infection in the past 6 months; (3) abnormal liver or renal function; (4) presence of viral infection or positive carrier status (hepatitis B virus, syphilis, and HIV). Finally, a total of 150 patients were registered for the analysis. The flow diagram of the study is shown in Supplementary Figure 1.

### 2.3. General Clinical Data Collection

Demographic and lifestyle data were obtained from the questionnaires, including age, gender, medication history, and family history of diabetes. According to the routine protocol, standardized measurement of body weight, height, and waist circumference was performed on every subject. The body mass index (BMI; weight/height^2^) was then calculated. Cushing's syndrome was screened by using the 24-hour urinary free cortisol or 1 mg overnight dexamethasone inhibition test. Following this, a low-dose dexamethasone suppression test was used to increase the diagnostic specificity for Cushing's syndrome. A complete evaluation of the pituitary-adrenal axis, 8-, 16-, and 24-h adrenocorticotropic hormone (ACTH) and cortisol levels, and serum cortisol levels were carried out. Plasma ACTH and cortisol levels were measured using a radioimmunoassay kit (Diasporic Inc., Stillwater, MN, USA). After overnight fasting for 12 hours, blood samples were collected for blood cell count and biochemical parameter detection (Japan Hitachi 7600 biochemical analyzer), respectively. Fasting samples, including plasma glycine hemoglobin (HbA1c) level, total cholesterol, and triglycerides, were obtained for routine determination by standard automated laboratory methods. After the baseline blood collection, a standard 75 g glucose tolerance test (OGTT) was carried out, and blood samples were collected both during fasting and 120 min after glucose-loading administration. The glucose oxidase method was used to evaluate the plasma glucose levels.

### 2.4. Statistical Analysis

Statistical Packages for Social Science (SPSS) software (version 13.0; SPSS, Chicago, IL, USA) was used for data analysis. Continuous variables of normal distribution were expressed as mean ± standard deviation. Nonnormal variables were expressed as median with an interquartile range. Analysis of variance (ANOVA) and Student's *t*-test were used to determine differences in means between groups. Bonferroni correction was applied for multiple comparisons. Nonnormally distributed values were log-transformed before analysis and were analyzed by Kruskal–Wallis 1-way ANOVA or Wilcoxon tests. The *χ*^2^-test was used to compare the rates. Partial Spearman's correlation analysis was performed to identify the correlation between leukocytes. Multiple linear regression analysis was used to detect independent correlations between blood leukocyte parameters and blood glucose levels. We also assessed the association of the blood leukocyte parameters with diabetes by multivariate logistic regression for different models. The specific effect of NEUT% on diabetes was further evaluated by dividing NEUT% into tertiles. A multivariate logistic regression model was used to obtain the adjusted odds ratio of diabetes. Patients with NEUT% <64.3 were defined as reference values. All statistical tests were two-tailed, and *p* < 0.05 was regarded as statistically significant.

## 3. Results

### 3.1. General Characteristics of the Study Population

**Figure 1:**
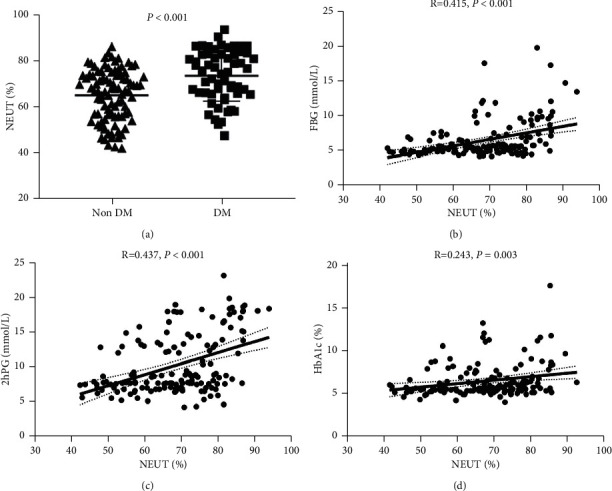
The percentage of neutrophils is associated with impaired glucose metabolism in patients with Cushing's syndrome. (a) Neutrophilic granulocyte percentage (NEUT%) levels are much higher in DM patients than in non-DM. (b) Fasting glucose (FBG) levels are positively correlated with the percentage of neutrophilic granulocyte. (c) Levels of 2-hour postprandial blood glucose (2hPG) are positively correlated with the percentage of neutrophilic granulocyte. (d) Glycated hemoglobin (HbA1c) is positively correlated with the percentage of neutrophilic granulocyte.

The demographics and baseline characteristics of the participants are displayed in Table 1. The mean age of the patients was 50.7 ± 13.8 years. Females were overrepresented in the study population compared to previous studies, with 31 (21%) of 150 cases being men and 119 (79%) being women. The number of female patients was nearly four times higher than that of male patients. Ignoring the gender difference, the average BMI was 25.0 ± 3.8 kg/m^2^. Again, among the 150 Cushing's syndrome patients, 58 patients (38.7%) were diagnosed with diabetes in our study. On average, in terms of baseline characteristics, patients with DM were older and had higher triglyceride levels (*p*=0.002). The following glycerin parameters were measured: fasting blood glucose (FBG) (*p* < 0.001), 2-hour postprandial blood glucose (2hPG) (*p* < 0.001), and HbA1c (*p* < 0.001) were higher in the diabetic group than in the nondiabetic group. Interestingly, patients with DM had significantly higher levels of 8 AM cortisol, 4 PM cortisol, and 12MN cortisol. In addition, we also found DM patients had higher WBC count level (7.8 ± 2.7 vs 6.7 ± 2.2) (*p*=0.008), neutrophil count (5.86 ± 2.6 vs 4.6 ± 2.1) (*p* < 0.001), and NEUT% (73.6 ± 11.1 vs 65.2 ± 11.1) (*p* < 0.001), as well as lower LYM% level (18.8 ± 9.7 vs 25.6 ± 9.5) (*p* < 0.001), whereas there was no significant difference in the lymphocyte count (Table 1). The mean NEUT% of DM patients was 73.6 ± 11.1, whereas that of non-DM patients was 65.2 ± 11.1 (*p* < 0.001) (Figure 1(a)).

### 3.2. Correlation between Blood Leukocyte Parameters and Blood Glucose Levels in Patients with Cushing's Syndrome

The probable relationship between white blood cells and glucose levels in patients with CS was further studied, and a scatter plot between NEUT% and glucose levels in 150 patients was developed. As shown in Figures 1(b)–1(d), NEUT% level was positively correlated with blood glucose parameters including FBG (*r* = 0.415, *p* < 0.001), 2hPG (*r* = 0.437, *p* < 0.001), and HbA1c (*r* = 0.243, *p*=0.005). In addition, strong correlations were established among the WBC, neutrophils, and NEUT% levels, ranging from 0.56 to 0.97 (data not shown). Furthermore, we also analyzed the correlation between other leukocyte parameters and blood glucose levels. As shown in Table 2, WBC was positively correlated with FBG (*r* = 0.168, *p* < 0.05) and 2hPG (*r* = 0.266, *p* < 0.01). Moreover, the neutrophil count was positively associated with FBG (*r* = 0.271, *p* < 0.01), 2hPG (*r* = 0.346, *p* < 0.01), and HbA1c (*r* = 0.160, *p* < 0.05). However, the correlation between blood lymphocyte parameters and FBG and 2hPG was negative, ranging from −0.274 to −0.418. No correlations were observed between the lymphocyte parameters and HbA1c.

As indicated in Table 1, serum cortisol levels in DM patients were considerably greater than those in nondiabetic patients, so we analyzed the correlation between cortisol levels and blood glucose levels. Positive correlations were found between serum cortisol levels and blood sugar parameters. 08 : 00 cortisol level was positively correlated with FBG (*r* = 0.358, *p* < 0.01), 2hPG (*r* = 0.410, *p* < 0.01), and HbA1c (*r* = 0.171, *p* < 0.05). Similar correlations were found in 16 : 00 cortisol with FBG (*r* = 0.353, *p* < 0.01) and 2hPG (*r* = 0.415, *p* < 0.01), as well as 24 : 00 cortisol with FBG (*r* = 0.346, *p* < 0.01) and 2hPG (*r* = 0.380, *p* < 0.01) (Table 2). These data demonstrate that blood glucose levels and cortisol levels are mutually influential.

NEUT% was an independent risk factor for the development of DM in Cushing's syndrome patients.

To determine independent risk factors for the development of DM, WBC, NEUT%, tertiles of NEUT%, LYM, and LYM% were entered in logistic regression analysis with enter selection in model 1 (Table 3). The whole analysis was divided into three models. In model 1, confounding factors were not adjusted. Compared with the lowest tertile of NEUT%, the risk of developing DM in the highest tertile of NEUT% was increased by 4.750 times (*p* < 0.001). After multivariate adjustments for gender, age, BMI, and triglyceride in model 2, NEUT% was still an independent risk factor (odds ratios (ORs) = 2.971; 95% CI, 1.733–4.977; *p* < 0.001) and the highest tertile of NEUT% increased by 8.452 folds compared with the lowest tertile of NEUT% (*p* < 0.001). The OR value of the highest tertile was higher than in the uncorrected model. Patients with higher triglyceride levels (OR = 1.852; 95% CI, 1.773–4.977; *p*=0.006) and age (OR = 1.048; 95% CI, 1.017–1.079; *p*=0.002) were also more likely to develop DM. Model 3 additionally corrected the 24 : 00 cortisol level based on model 2. The NEUT% tertile remained as a major risk factor for the development of DM independent of gender, age, BMI, 24 : 00 cortisol, and triglyceride level in CS patients (*p*=0.004). The highest tertile of NEUT% increased by 6.486 folds compared with the lowest tertile of NEUT% (*p*=0.004). Similarly, patients with higher triglyceride levels (OR = 1.856; 95% CI, 1.198–2.875; *p*=0.006) and age (OR = 1.049; 95% CI, 1.018–1.081; *p*=0.002) had a higher risk of developing DM. These results suggest that NEUT% is an independent risk factor for DM in CS patients. To further compare blood leukocyte levels (WBC, NEUT, NEUT%, LYM, LYM%, and NLR) in patients with and without DM, a univariate analysis of variance (UNIANOVA) model was used. Adjustments were made for gender, age, BMI, and TG. The UNIANOVA model showed that NEUT% (*F* = 11.796, *p* < 0.001) remained a significant predictor of DM, and the highest tertile of NEUT% was strongly positively associated with the risk of diabetes (95% CI: 0.244–0.604; *p* < 0.001) (Supplementary Table 1).

## 4. Discussion

Glucose intolerance and diabetes mellitus are widespread in Cushing's syndrome patients [19]. It has been shown that the prevalence of DM in CS ranges between 20% and 50% [1]. The current study illustrates that the prevalence of diabetes in Cushing's syndrome was 38.7%. From the demographic characteristics of our research subjects, the participants were all middle-aged and elderly people. Older people may be associated with uncomplicated diseases that come with increasing age, and the prevalence of DM was relatively high. In addition, in the correlation analysis, we also found a close correlation between cortisol levels and glucose. These findings confirm those of previous studies. With reference to the previously reported literature, we found that the fasting glucose level of patients with active CS was generally higher than that in the remission period. A certain degree of abnormal glucose metabolism is also present in patients with active CS [20]. Furthermore, some articles suggest that elevated fasting glucose may be a feature of subclinical CS. There was a definite correlation between fasting plasma glucose and exogenous glucocorticoid levels, and it was found that the two factors interact with each other [21]. In an age- and gender-matched cohort study, it was also demonstrated that the glucose level of patients in the CS group was significantly higher than in the control group. Still, the above studies only described the phenomenon and did not provide in-depth insight into the pathogenesis. Therefore, the precise mechanism of hyperglycemia in CS patients was discussed. Several reports on pathophysiological mechanisms underline that glucocorticoids can induce insulin resistance, leading to the inhibition of the insulin signaling pathway. Glucose cannot be effectively absorbed and utilized, resulting in the onset of DM in patients with CS [22–24]. In addition, it has also been reported that glucocorticoids can directly and indirectly affect insulin secretion and cause impairment of pancreatic *β*-cell function. In vivo studies have revealed a compensation mechanism for the response to glucocorticoid exposure. Studies inferred that high glucocorticoid levels in CS patients led to more insulin secretion and stress-induced increase of blood glucose [25, 26]. However, in vitro studies have also shown that the direct inhibition of insulin secretion may be explained by the fact that glucocorticoids can directly downregulate the RNA level of transcription factors required for the secretion process in response to cytoplasmic Ca^2 +^ [27–29]. We also found some clues in population studies. A population-based study was conducted by Kamba et al., where the relation between *β*-cell function and cortisol was investigated. It was found that decreased insulin secretion and glucocorticoids-inhibited *β*-cell function were linked to higher cortisol levels (*p*=0.03) [24]. In general, CS led to impaired glucose metabolism mainly by reducing insulin action and reducing glucose treatment [30–32]. At present, further research is required to understand the effect of excessive cortisol on blood glucose.

As can be seen in our baseline characteristics data, the number of WBCs was considerably higher in DM patients. We first discuss the reasons for the elevated WBC in CS patients. Glucocorticoid-induced leukocytosis has been attributed to a variety of mechanisms, including increased PMN release from the bone marrow to the circulation [33], which elevated blood leukocytes. In addition, delayed apoptosis of circulating neutrophils [34], accumulation in the blood, and decreased cell migration from the blood vessels into peripheral tissues have been reported [35]. These pathophysiological processes all lead to an increasing number of blood leukocytes in patients with CS. Reviewing the literature, we have found that stress signals such as cortisol can activate mature granulocytes in blood for rapid desalination, resulting in rapid elevation of blood leukocytes. Clinical studies have provided evidence that intravenous or oral dexamethasone on healthy people caused an increased neutrophil count and lymphopenia, with a linear increment with intravenous dose but not with oral dose [36, 37]. Dexamethasone is considered as a reflection of the stress that directly stimulates leukocytes when administered intravenously, whereas oral dexamethasone goes through the first-pass elimination. Dexamethasone is digested before intestinal absorption without directly affecting leukocytes. Cushing's syndrome should be a part of the routine examination of patients with unexplained leukocytosis, as revealed in this series. However, a direct relationship between leukocytosis and disturbed glucose metabolism in CS patients has not been found previously.

Type 2 diabetes features a chronic, low-grade inflammation, and generalized activation of the innate immune system is an essential component of its pathophysiology [38]. The increased inflammatory cells in DM patients can be partly explained. The total number of peripheral leukocytes has been shown to be related to diabetes risk in numerous studies [39, 40]. Several hemogram markers are independent risk factors and predictors of the occurrence and development of diabetes and its complications, which have become research hotspots. Previous articles have reported that RDW [12] reflects high levels of oxidative stress and chronic inflammation and is a marker for evaluating the prognosis of diabetic patients. MPV [13] is closely related to diabetic microangiopathy; PLR [15] has also been considered an emerging indicator of inflammation in recent years, but its relationship with diabetes is still controversial. In this study, we focused mainly on the effect of leukocyte components in blood cells on diabetes. Therefore, in the Results section, parameters such as erythrocyte and platelets were not described. Future studies can systematically relate the effects of each cell component on diabetes. As for NLR, although it is reported in the literature that increased NLR is closely related to diabetic cardiovascular disease, some studies have shown that NLR is not predictive of elevated glucose [14, 41]. NLR is the ratio of neutrophil count to lymphocyte count. As there is no difference in the lymphocyte count, the effect of neutrophils on diabetes may not be apparent with NLR as a disease predictor. Therefore, NLR is not as effective as neutrophils in predicting the occurrence and outcome of diabetes. After matching possible confounding factors in this study, only the OR of NEUT% was statistically different. These results support the key pathological role of innate immune cells in the development of diabetes. Although WBC is considered to be able to predict the incidence of T2DM, the data on how WBC subtypes are associated with T2DM risk are notably limited and conflicting [42, 43]. As a marker of subclinical inflammation, leukocytes are directly associated with insulin resistance and inversely correlated with insulin secretion. Leukocytes have also been shown to inhibit insulin secretion [44, 45]. The insulin signaling pathway is intercepted by the elevated number of white blood cells in the whole body. Again, neutrophils appear to be the immune cells involved in the proinflammation procedure that underlies insulin resistance [46]. Increased neutrophil counts can mediate insulin resistance by enhancing inflammation. Our results were in agreement with these reports. We found that the number of WBCs was significantly increased in DM people. In the correlation analysis, all leukocyte parameters were strongly and positively correlated with blood glucose parameters, especially FBG and 2hPG. The present cross-sectional study of a population with Cushing's syndrome showed that NEUT% was independently associated with diabetes. Participants with elevated NEUT% had significantly greater odds of developing diabetes than other individuals. Our findings suggest that NEUT% count can be seen as a predictor of diabetes in CS patients. NEUT% is a major independent risk factor for DM progress in CS patients. After adjusting for other confounders, the OR of the highest NEUT% tertile was still more than 6-fold higher. Taken in conjunction, the present results on the effects of white blood cells were significantly associated with diabetes, which may be mediated by the combination of insulin resistance and *β*-cell dysfunction.

Nevertheless, there were some limitations to this study. The present study was a cross-sectional study design and lacked follow-up data. Therefore, a causal relationship of NEUT% levels and diabetes cannot be determined in this Chinese Cushing's syndrome population. The sample size was not estimated before the study and was small. Furthermore, the current findings are based on a hospital-based study. Thus, a future prospective cohort study in population-based samples is necessary to elucidate this relationship further. There is also a lack of mechanistic insight into the potential pathophysiological role of neutrophils in DM progress in this clinical study.

## 5. Conclusion

In conclusion, the present study demonstrated that the prevalence of diabetes in Cushing's syndrome was 38.7%. Furthermore, increased NEUT% levels were closely associated with worsening diabetes in a Chinese Cushing's syndrome population. These results may offer new insights into the pathophysiology of blood leukocyte parameters on the regulation of diabetes.

## Figures and Tables

**Table 1:** General characteristics of Cushing's syndrome patients with or without diabetes.

Variables	All (*n* = 150)	Non-DM (*n* = 92)	DM (*n* = 58)	*p* value
Age (years)	50.7 ± 13.8	48.4 ± 14.3	54.2 ± 12.2	0.012
Gender (M/F)	31/119	15/77	16/42	0.300
BMI (kg/m^2^)	25.0 ± 3.8	25.0 ± 3.9	25.1 ± 3.7	0.934
Triglyceride (mmol/L)	1.7 ± 1.3	1.4 ± 0.9	2.1 ± 1.6	0.002
FBG (mmol/L)	6.3 ± 2.7	5.0 ± 0.6	8.4 ± 3.4	<0.001
2hPG (mmol/L)	10.3 ± 4.3	7.4 ± 1.2	15.0 ± 3.4	<0.001
HbA1c (%)	6.6 ± 2.0	5.5 ± 0.4	8.3 ± 2.3	<0.001
8 AM cortisol (nmol/L)	543.9 (378.3–762.8)	510.8 (348.9–689.7)	641.4 (436.1–889.6)	0.002
4 PM cortisol (nmol/L)	473.2 (287.3–651.8)	413.7 (219.3–606.8)	544.4 (324.0–866.1)	0.002
12 MN corstisol (nmol/L)	396.9 (211.3–578.9)	357.1 (175.9–553.8)	458.2 (289.0–703.1)	0.008
LDDST cortisol (nmol/L)	232.0 (128.0–484.0)	288.0 (139.0–576.0)	147.0 (123.0–410.0)	0.230
WBC (×10^9^)	7.1 ± 2.5	6.7 ± 2.2	7.8 ± 2.7	0.008
Neutrophils (×10^9^)	5.1 ± 2.4	4.6 ± 2.1	5.86 ± 2.6	<0.001
NEUT%	68.4 ± 11.8	65.2 ± 11.1	73.6 ± 11.1	<0.001
Lymphocytes (×10^9^)	1.5 ± 0.6	1.6 ± 0.4	1.4 ± 0.7	0.055
LYM%	23.0 ± 10.1	25.6 ± 9.5	18.8 ± 9.7	<0.001
Scr (*μ*mol/L)	67.4 ± 36.8	66.3 ± 35.3	69.2 ± 39.1	>0.05
eGRF (ml/min/1.73 m^2^)	91 ± 25	93 ± 22	86 ± 29	>0.05
TC (mmol/L)	5.06 ± 1.55	4.94 ± 1.00	5.26 ± 2.13	>0.05
LDL-C (mmol/L)	2.86 ± 0.95	2.85 ± 0.89	2.87 ± 1.03	>0.05
HDL-C (mmol/L)	1.38 ± 0.41	1.45 ± 0.38	1.25 ± 0.42	>0.05

Data are means ± SD, median (interquartile range), and log-transformed for the *t*-test. BMI, body mass index; FBG, fasting blood glucose; 2hPG, 2-hour postprandial blood glucose; HbA1c, glycosylated hemoglobin; WBC, white blood cell; NEUT%, neutrophilic granulocyte percentage; LYM%, lymphocyte percentage; Scr, serum creatinine; eGRF, estimated glomerular filtration rate; TC, total cholesterol; LDL-C, low-density lipoprotein cholesterin; HDL-C, high-density lipoprotein cholesterin.

**Table 2:** Correlation analysis related to glucose homeostasis.

	FBG	2hPG	HbA1c
WBC	0.168^*∗*^	0.266^*∗∗*^	0.122
Neutrophils	0.271^*∗∗*^	0.346^*∗∗*^	0.160^*∗*^
NEUT%	0.415^*∗∗*^	0.437^*∗∗*^	0.243^*∗*^
Lymphocyte (×10^9^)	−0.351^*∗*^	−0.274^*∗*^	−0.93
LYM%	−0.395	−0.418^*∗*^	−0.201
8 AM cortisol (nmol/L)	0.358^*∗∗*^	0.410^*∗∗*^	0.171^*∗*^
4 PM cortisol (nmol/L)	0.353^*∗∗*^	0.415^*∗∗*^	0.151
12 MN cortisol (nmol/L)	0.346^*∗∗*^	0.380^*∗∗*^	0.105

^*∗∗*^Correlation is significant at the 0.01 level (2-tailed); ^*∗*^correlation is significant at the 0.05 level (2-tailed).

**Table 3:** Logistic regression analysis used to determine the risk factors for development of DM.

	Model 1		Model 2		Model 3	
	OR (95% CI)	*p* value	OR (95% CI)	*p* value	OR (95% CI)	*p* value
NEUT%	**2.228 (1.440–3.448)**	**<0.001**	**2.971 (1.773–4.977)**	**<0.001**	**2.542 (1.337–4.835)**	**0.004**
Tertile1	1.000		1.000		1.000	
Tertile2	1.490 (0.618–3.592)	0.374	1.677 (0.621–4.528)	0.308	1.512 (0.534–0.281)	0.437
Tertile3	**4.750 (2.008–11.236)**	**<0.001**	**8.452 (3.057–23.367)**	**<0.001**	**6.486 (1.802–23.339)**	**0.004**
WBC	NS	NS	NS	NS	NS	NS
NEUT	NS	NS	NS	NS	NS	NS
LYM	NS	NS	NS	NS	NS	NS
LYM%	NS	NS	NS	NS	NS	NS
Gender	—	—	NS	NS	NS	NS
Age (years)	—	—	**1.048 (1.017–1.079)**	**0.002**	**1.049 (1.018–1.081)**	**0.002**
BMI (kg/m^2^)	—	—	NS	NS	NS	NS
Triglyceride (mmol/L)	—	—	**1.852 (1.773–4.977)**	**0.006**	**1.856 (1.198–2.875)**	**0.006**
12 MN cortisol (nmol/L)	—	—	—	—	NS	NS

NS: not significant; model 1: WBC, NEUT, NEUT%, LYM, and LYM% were included; model 2: WBC, NEUT, NEUT%, LYM, and LYM% were included, adjusted for gender, age, BMI, and triglyceride; Model 3: WBC, NEUT, NEUT%, LYM, and LYM% were included, adjusted for gender, age, BMI, triglyceride, and 12 MN cortisol. Bold values indicate all indicators with statistical differences *p* < 0.05.

## Data Availability

The data supporting this study's findings are not publicly available but are available from the corresponding author upon reasonable request.

## Abbreviations

CS: Cushing's syndrome
DM: Diabetes mellitus
WBC: White blood cell
NEUT%: Neutrophilic granulocyte percentage
LYM%: Lymphocyte proportion
FBG: Fasting blood glucose
2hPG: 2-hour postprandial blood glucose
GC: Glucocorticoid
CD: Cushing's disease
T2DM: Type 2 diabetes
PMN: Polymorphonuclear
eGFR: Estimated glomerular filtration rate
BMI: Body mass index
ATCH: Adrenocorticotropic hormone
HbA1c: Glycated hemoglobin
OGTT: Oral glucose tolerance test
RDWred: Blood cell distribution width
MPV: Mean platelet volume
NLR: Neutrophil count to lymphocyte count ratio
PLR: Platelet count to lymphocyte count ratio
MLR: MPV to lymphocyte count ratio.

## Supplementary Materials

Supplementary Materials Supplementary Figure 1: flow diagram of the study. Supplementary Table 1: associations between predictors and diabetes mellitus (UNIANOVA analysis).
