# Supplementary material for: Association between Neutrophilic Granulocyte Percentage and Diabetes Mellitus in Cushing's Syndrome Patients: A Cross-Sectional Study
Source: Int J Endocrinol. 2021 Nov 29;2021:9536730. doi: 10.1155/2021/9536730 (PMC8648481; doi:10.1155/2021/9536730)
Supplement: Supplementary Materials — Supplementary Figure 1: flow diagram of the study. Supplementary Table 1: associations between predictors and diabetes mellitus (UNIANOVA analysis). [file 9536730.f1.docx]

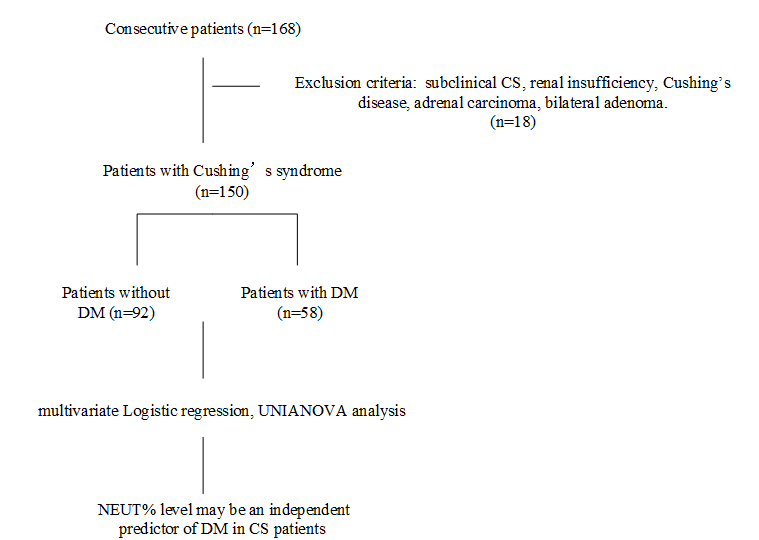


Supplementary figure 1: Flow diagram of the study.

**Supplementary table 1. Associations between predictors and diabetes mellitus (UNIANOVA analysis)**

| predictors | DM | | | | F | p-value |
| --- | --- | --- | --- | --- | --- | --- |
|  | Non-DM(n=92) | | DM (n=58) | |  |  |
|  | count | % | count | % |  |  |
| WBC |  |  |  |  | 4.522 | 0.012 |
| Tertile1 | 38 | 76.00% | 12 | 24.00% |  |  |
| Tertile2 | 27 | 54.00% | 23 | 46.00% |  | 0.014 |
| Tertile3 | 27 | 54.00% | 23 | 46.00% |  | 0.007 |
| NEUT |  |  |  |  | 7.684 | 0.001 |
| Tertile1 | 40 | 80.00% | 10 | 20.00% |  |  |
| Tertile2 | 25 | 50.00% | 25 | 50.00% |  | 0.001 |
| Tertile3 | 27 | 54.00% | 23 | 46.00% |  | 0.001 |
| **NEUT%** |  |  |  |  | **11.796** | **<0.001** |
| **Tertile1** | **38** | **76.00%** | **12** | **24.00%** |  |  |
| **Tertile2** | **34** | **68.00%** | **16** | **32.00%** |  | **0.232** |
| **Tertile3** | **20** | **40.00%** | **30** | **60.00%** |  | **<0.001** |
| LYM |  |  |  |  | 12.090 | <0.001 |
| Tertile1 | 19 | 38.00% | 31 | 62.00% |  |  |
| Tertile2 | 39 | 78.00% | 11 | 22.00% |  | <0.001 |
| Tertile3 | 34 | 68.00% | 16 | 32.00% |  | <0.001 |
| LYM% |  |  |  |  | 8.073 | <0.001 |
| Tertile1 | 21 | 42.00% | 29 | 58.00% |  |  |
| Tertile2 | 33 | 66.00% | 17 | 34.00% |  | 0.008 |
| Tertile3 | 38 | 76.00% | 12 | 24.00% |  | <0.001 |
| NLR |  |  |  |  | 10.433 | <0.001 |
| Tertile1 | 39 | 78.00% | 11 | 22.00% |  |  |
| Tertile2 | 33 | 67.30% | 16 | 32.70% |  | 0.096 |
| Tertile3 | 20 | 39.20% | 31 | 60.80% |  | <0.001 |

%: [N count/N count (Non-DM +DM)]*100%
